# Speech Spectrum's Correlation with Speakers' Eysenck Personality Traits

**DOI:** 10.1371/journal.pone.0033906

**Published:** 2012-03-16

**Authors:** Chao Hu, Qiandong Wang, Lindsey A. Short, Genyue Fu

**Affiliations:** 1 Department of Psychology, Zhejiang Normal University, Jinhua, Zhejiang Province, China; 2 Department of Psychology, Brock University, St. Catharines, Ontario, Canada; 3 Hangzhou College for Kindergarten Teachers, Zhejiang Normal University, Hangzhou, Zhejiang Province, China; University of Leicester, United Kingdom

## Abstract

The current study explored the correlation between speakers' Eysenck personality traits and speech spectrum parameters. Forty-six subjects completed the Eysenck Personality Questionnaire. They were instructed to verbally answer the questions shown on a computer screen and their responses were recorded by the computer. Spectrum parameters of /sh/ and /i/ were analyzed by Praat voice software. Formant frequencies of the consonant /sh/ in lying responses were significantly lower than that in truthful responses, whereas no difference existed on the vowel /i/ speech spectrum. The second formant bandwidth of the consonant /sh/ speech spectrum was significantly correlated with the personality traits of Psychoticism, Extraversion, and Neuroticism, and the correlation differed between truthful and lying responses, whereas the first formant frequency of the vowel /i/ speech spectrum was negatively correlated with Neuroticism in both response types. The results suggest that personality characteristics may be conveyed through the human voice, although the extent to which these effects are due to physiological differences in the organs associated with speech or to a general Pygmalion effect is yet unknown.

## Introduction

In his classic book *The Expression of the Emotions in Man and Animals*, Darwin states, “With many kinds of animals, man included, the vocal organs are efficient in the highest degree as a means of expression” [1]. Indeed, numerous studies have suggested that we can use acoustic characteristics to perceive speakers' emotional states [2], [3]. For example, both fundamental and formant frequencies as well as durations of speech utterances are thought to be sufficient to communicate a wide variety of emotions [4] and some researchers have even concluded that the musical expression of emotion is due to the acoustic pattern similarity between music and the human voice [5].

Just as musical instruments are allocated with different characters by their timbres, individuals also tend to believe that speakers' speech characteristics are correlated with their personality traits. It has long been known that listeners' judgments of speakers' personality traits are influenced by speakers' speech characteristics such as intensity, clearness, flow of speech, and poise [6]. For example, the intensity of speech seems to particularly affect listeners' perceptions of speakers' extraversion [7]. Women tend to believe that men with faster speech have higher scores on the Big Five personality factors of openness and extraversion [8]. Moreover, candidates' speech characteristics affect interviewers' ratings of their personalities in employment interviews [9]. Indeed, the Big Five personality factors of agreeableness and conscientiousness are found to vary in a pattern related to levels of vocal attractiveness such that both personality factors predict performance more strongly for people with more attractive voices [10]. Some researchers have argued that voice characteristics alone are sufficient to elicit moderately accurate impressions of speakers' personality traits; even when listeners do not understand the speakers' language, they are able to assess speakers' personalities accurately to a degree [11].

In summary, individuals tend to believe that physical traits of speech are correlated with speakers' personalities. This impression seems reasonable, given that speech spectrum has repeatedly been shown to be correlated with emotion [2], [3] and emotion is considered to be coherent with personality just as weather is coherent with climate [12]. Yet very few studies have examined the correlation between speakers' personalities and the actual physical characteristics of speech. Research has demonstrated that speakers' voice type (loud-slow, loud-fast, soft-slow, soft-fast) is associated with their personality [13]; however, speed and volume are not intrinsic to speech timbre and are easy for speakers to deliberately change. Nesic [14] reported that speakers' fundamental frequency of the vowels /a/ and /i/ spoken in a calm emotional state is significantly correlated with several factors of their Tridimensional personality test results. However, although formant frequency and bandwidth have been shown to be correlated with timbre and the vocal communication of emotion [15], these factors were not analyzed in Nesic's research.

Previous work in our lab [16] demonstrated that speakers' 16 PF traits were significantly correlated with the formant parameters of the consonant /sh/. Participants completed the 16 PF Questionnaire and were then given false feedback on the results of their test (positive, negative, or neutral). After receiving this feedback, they were asked to verbally describe their feelings at the time. Their responses during different emotional states (positive, negative, and neutral) were recorded, and the frequency and bandwidth of the former three formants of the consonant /sh/ in all responses were analyzed by Praat voice software. The spectrum parameters of the consonant /sh/ differed based on the emotional state expressed such that the first formant frequency F1 and the third formant frequency F3 significantly increased when participants experienced positive emotions relative to neutral or negative emotions. Additionally, across all emotional states, the correlation between formant parameters and personality traits was significant, especially between the third formant frequency of the consonant /sh/ and participants' emotional sensitivity and between the first formant bandwidth and participants' social boldness.

Although these results suggest a relationship between personality and the speech spectrum parameters of the consonant /sh/, it is unclear whether the demonstrated correlations were significant simply because of the relationship between participants' personality traits and their emotional reactions. The majority of the responses analyzed in our previous research were taken from emotionally charged speech. Thus, in the current study, it was necessary to examine whether the correlation between speakers' personality traits and speech spectrum parameters continued to hold in the absence of induced positive and negative emotion.

The current study was designed to extend our previous research by examining the correlation between speakers' Eysenck personality trait scores and the speech spectrum parameters of the consonant /sh/ and the vowel /i/ in participants' verbal responses to the questionnaire. We chose to use the Eysenck Personality Questionnaire rather than the 16 PF Questionnaire from our previous study because the Eysenck questionnaire requires participants to provide simple “yes” and “no” responses that can be easily recorded and analyzed. Furthermore, we elected to analyze the speech spectrum parameters of both the consonant /sh/ and the vowel /i/ in the present study because it has been previously demonstrated that the nature of consonant and vowel phonemes differs in that the vocal cords vibrate only when vowels are spoken [17]. We hypothesized that the correlation between speakers' personality traits and formant parameters may be affected by the individual characteristics of their vocal organ positions and movements during speech; thus, the correlation between personality traits and speech spectrum may differ across consonant and vowel vocalizations.

In the present study, participants were instructed to verbally answer questions from the Eysenck Personality Questionnaire (EPQ). Their responses were divided into four categories: truthful /shi/ (“yes”), lying /shi/ (“yes”), truthful /bushi/ (“no”), and lying /bushi/ (“no”). If a response was scored as a socially desirable lie by the L scale grading standard, then it was classified as lying. We made this classification for two reasons. First, previous research in our lab demonstrated that the speech spectrum parameters of the consonant /sh/ in /bushi/ and /shi/ are different [16]. Second, Ekman, Friesen, and Scherer [18] have suggested that there are differences in vocal pitch between truthful and lying speech; thus the correlation may vary between these two speech patterns.

Participants' responses were recorded and analyzed by Praat 5.1.30 software. Measured speech spectrum parameters included fundamental frequency, frequency of the former three formants, and bandwidth of the first three formants. Formants are the regions in the spectrogram in which the amplitude of the acoustic energy is high. They are influenced by the length or dimensions of the vocal tract within or around the larynx [19] and reflect the natural resonance frequencies of the vocal tract, which are not static but can be changed by altering vocal tract shape such as by adjusting the relative position of the jaw or tongue [20]. Formant frequency and bandwidth, particularly the frequency of the former three formants, have been suggested to be extremely important in perceiving speakers' personalities [21], and thus we chose to focus our investigation on these two characteristics of human speech.

## Method

### Ethics Statement

All procedures used in the current study were approved by the Ethics Committee of Zhejiang Normal University. Participants provided oral consent prior to testing and were ensured that no harm would come to them through their participation in the experiment. They were told that they would complete a personality test and that their vocal responses would be recorded and analyzed. Results of the personality test and vocal responses were kept private, and participants were told that they had the option to quit at any time during the experiment and still receive monetary payment.

### Participants

Forty-six undergraduate students (22 male, aged 18–23) at Zhejiang Normal University, Jinhua, Zhejiang Province of China volunteered to participate in the experiment. All participants had never completed the Eysenck Personality Questionnaire before and possessed clear pronunciation of the Chinese language. Participants received a small payment following the completion of the experiment.

### Materials

Participants completed The Revised Eysenck Personality Questionnaire Short Scale for Chinese edited by Zhonggeng Chen, which has been demonstrated to be reliable and valid for Chinese participants [22]. The inventory contains four personality trait scales: Psychoticism (P), Extraversion (E), Neuroticism (N), and Social Desirability (L). The L scale contains questions on which individuals tend to lie for social desirability, and the scoring standard is the same for everyone. If a participant received a score of one instead of zero on an L scale question, then this response was recorded as a lie. Praat 5.1.30 software in a Windows 2003 operating system was used to analyze participants' recorded voice samples.

### Procedure

Participants individually completed the questionnaire in a quiet room. They were instructed to answer all Eysenck personality questions shown on a computer screen one after another by speaking “yes” or “no” (/shi/ or /bushi/ in Chinese) in front of a microphone. Their responses were recorded by the computer. Completion of the personality inventory took approximately 15 minutes.

**Figure 1:**
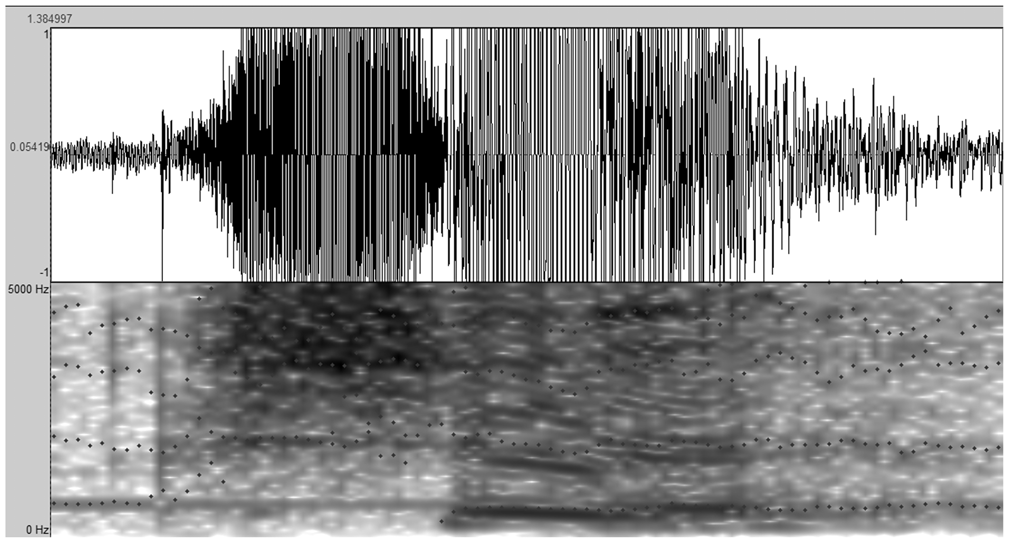
Sample sonogram as provided by Praat 5.1.30 software.

Voice samples that were recorded during participants' responses to the Social Desirability scale were then analyzed in Praat 5.1.30 software. We analyzed only the speech spectrum of /sh/ and /i/ in utterances of /shi/ or /bushi/ (“yes” or “no”). Across all participants, there were 141 lying /shi/ responses, 296 truthful /shi/ responses, 334 lying /bushi/ responses, and 133 truthful /bushi/ responses. In our analysis, we first selected the consonant and vowel segments from each of the utterances according to the sonograms provided by the software (as shown in Figure 1). We then analyzed the consonant /sh/ and the vowel /i/ separately using the following spectrum parameters: fundamental frequency; frequency of the former three formants: F1, F2, and F3; and bandwidth of the first three formants: B1, B2, and B3.

## Results

### EPQ Results

The mean values and associated standard deviations of the 46 participants' personality trait scores were as follows: Extraversion: 11.39 (*SD* = 5.00), Neuroticism: 12.07 (*SD* = 5.17), Psychoticism: 4.85 (*SD* = 2.48), and Social Desirability: 10.65 (*SD* = 3.25). Psychoticism was significantly positively correlated with Neuroticism, *r* = 0.29, *p*<.05. Social Desirability was negatively correlated with both Neuroticism, *r* = −0.39, *p*<.01, and Psychoticism, *r* = −0.45, *p*<.01. All other correlations did not reach significance, *p*s>.05.

### Speech Spectrum Parameters for Truthful versus Lying Speech

The mean values and associated standard deviations of the speech spectrum parameters of /sh/ and /i/ are shown in Tables 1 and 2. Across all participants, paired sample *t*-tests revealed that the frequency of the third formant (F3) of /sh/ in lying /shi/ responses was significantly lower than that in truthful /shi/ responses, *t*(45) = −2.22, *p*<.05. Furthermore, frequencies of the former three formants of /sh/ in lying /bushi/ responses were all significantly lower than those in truthful /bushi/ responses, *df* = 45, all *t*s<−2.14, all *p*s<.05. However, there was no significant difference between the speech spectrum parameters of /i/ in truthful and lying /shi/ responses or in truthful and lying /bushi/ responses, *df* = 45, all *t*s<1.67, all *p*s>.10.

**Table 1:** Mean Values and Standard Deviations of /Sh/ Speech Spectrum Parameters (Unit: Hz).

	Truthful “yes”/shi/	Lying “yes”/shi/	Truthful “no” /bushi/	Lying “no” /bushi/
F1	2028 (306)	1988 (289)	2041 (352)	1994 (324)
F2	3393 (349)	3360 (364)	3412 (409)	3344 (372)
F3	4529 (214)	4479 (237)	4606 (258)	4551 (246)
B1	949 (508)	1062 (624)	985 (731)	984 (509)
B2	875 (577)	757 (489)	697 (419)	743 (323)
B3	740 (211)	790 (379)	873 (480)	887 (294)

*Note*. F1 = first formant frequency, F2 = second formant frequency, F3 = third formant frequency. B1 = first formant bandwidth, B2 = second formant bandwidth, B3 = third formant bandwidth.

**Table 2:** Mean Values and Standard Deviations of /I/ Speech Spectrum Parameters (Unit: Hz).

	Truthful “yes” /shi/	Lying “yes” /shi/	Truthful “no” /bushi/	Lying “no” /bushi/
F1	957 (284)	957 (297)	1038 (332)	1021 (290)
F2	2540 (326)	2563 (329)	2569 (344)	2586 (322)
F3	4007 (338)	4044 (333)	4137 (370)	4111 (345)
B1	678 (274)	719 (310)	797 (417)	754 (253)
B2	815 (402)	769 (476)	861 (647)	784 (377)
B3	797 (473)	844 (475)	732 (330)	818 (330)

*Note*. F1 = first formant frequency, F2 = second formant frequency, F3 = third formant frequency. B1 = first formant bandwidth, B2 = second formant bandwidth, B3 = third formant bandwidth.

### Correlations Between Personality Trait Scores and Speech Spectrum Parameters

For both truthful and lying responses, we calculated the Pearson correlation between personality trait scores (Extraversion, Neuroticism, Psychoticism) and speech spectrum parameters. Social Desirability was not included in this analysis because we assumed it was better not to use the speech samples from the Social Desirability scale to analyze the relationship between the scale's score and speech spectrum.

In truthful /shi/ responses, the second formant bandwidth (B2) of the consonant /sh/ was positively correlated with Neuroticism, *r* = 0.34, *p*<.05, and Psychoticism, *r* = 0.36, *p*<.05. In truthful /bushi/ responses, B2 of the consonant /sh/ was negatively correlated with Extraversion, *r* = −0.34, *p*<.05. Additionally, the third formant bandwidth of the consonant /sh/ was positively correlated with Extraversion, *r* = 0.30, *p*<.05. In lying /shi/ responses, the second formant bandwidth (B2) of the consonant /sh/ was significantly correlated with Extraversion, *r* = −0.30, *p*<.05, and Psychoticism, *r* = 0.32, *p*<.05. In lying /bushi/ responses, B2 was positively correlated with Psychoticism, *r* = 0.34, *p*<.05. In both lying and truthful /shi/ and /bushi/ responses, the first formant frequency of /i/ was significantly negatively correlated with Neuroticism, all *r*s<−0.31, *p*<.05. Moreover, the third formant bandwidth of /i/ was significantly negatively correlated with Neuroticism in lying /bushi/ responses, *r* = −0.31, *p*<.05. All other correlations did not reach significance, *p*s>.05.

## Discussion

The current study demonstrated that there are a number of significant correlations between voice characteristics and personality traits. To our knowledge, this is the first paper to report that Eysenck personality trait scores were significantly correlated with the speech spectrum parameters of formant frequency and bandwidth. Moreover, the correlations varied between different phoneme types (consonant or vowel) and response types (/shi/ or /bushi/, lying or truthful). Mainly, the first formant frequency of the vowel /i/ was significantly correlated with Neuroticism in all response types, whereas the significant correlation between the second formant bandwidth of the consonant /sh/ and the personality traits of Psychoticism, Extraversion, and Neuroticism differed across lying/truthful and affirmative/negative responses (e.g., Neuroticism was significantly correlated with the second formant bandwidth only in truthful affirmative /shi/ responses). The difference in the relationship between speech spectrum and personality traits for consonants and vowels may be because of the difference between the vocal tract dimensions that affect formant parameters; the vocal shapes during the phonation of /sh/ and /i/ are quite different from one another and the vocal cords vibrate only during the phonation of /i/ [17]. This may also account for the difference in the lying effect on the speech spectrums of /sh/ and /i/. That is, formant frequencies of the consonant /sh/ in lying responses were significantly lower than that in truthful responses, while no lying effect existed on the speech spectrum of the vowel /i/.

As shown in our results, the correlations between various personality traits and formant bandwidth are diverse, while those with formant frequency are identical. This pattern of results may be due to the different factors that affect formant parameters in human speech. That is, formant bandwidth is determined by variant factors, such as vocal tract wall friction and the opening and pressure drop at the glottis [23] and posterior glottal opening [24]. The diverse correlations between the bandwidth of the consonant /sh/ and personality traits across different response types may be caused by individual differences in these factors during phonation when participants are prompted with different kinds of questions. On the other hand, formant frequencies are determined by the shape and length of the vocal tract [25], which are more stable during phonation of the vowel /i/. The significant correlation between Neuroticism and the first formant frequency of the vowel /i/ may reflect a relationship between vocal organ chamber length and Neuroticism, just as several attributes of the human face appear to be related to an individual's behavioral disposition (e.g., facial width-to-height ratio as an indicator of male aggression [26]). Our results provide compelling evidence for the idea that personality traits may be communicated through the speech spectrum, just as such traits may be displayed in the characteristics of the human face [27], [28].

Similar to the results of our past research using the 16PF questionnaire [16], the correlation between personality trait scores and speech spectrum parameters of the consonant /sh/ was found to vary across response types. However, unlike Nesic [14], we did not find a significant correlation between fundamental frequency and personality traits, which may be due to the difference in the vocal samples used in the two studies. Whereas Nesic analyzed single meaningless vowel sounds in his sample of participants, we analyzed the vowels from meaningful and natural responses, a more ecologically valid paradigm.

In summary, the current study demonstrates that perceiving others' personality characteristics through their voice in natural situations is possible, and distinguishing among phoneme (consonant and vowel) and response types (lying and truthful, affirmative and negative) is crucial in order to do so. However, it is still unclear whether the correlations between personality traits and formant parameters are due to the physical traits of speakers' vocal tracts during phonation or to listeners' perceptions of speakers' personality characteristics based on their vocal attributes, which in turn creates a Pygmalion effect. It has been repeatedly demonstrated that teachers' expectations of students affect students' academic performance, which subsequently serves to confirm teachers' previous expectations [29]. Similarly, people who are born with certain speech characteristics may be perceived to possess certain personality traits that they may not actually have; however, over time, because others expect them to possess these traits and interact with them in a way that reflects this assumption, individuals will begin to behave in a way that is consistent with these expectations. Future research should further explore such self-fulfilling prophecy effects and examine whether changes in behavior occur over time as individuals are confronted with expectations about their personality based on their voice type. For example, will individuals' personalities dramatically change when their speech formants are altered by a vocal organ disease and how might this occur?

With the development of voice recording and analysis technology, systematic classification and analysis of a large amount of vocal samples will become easier and more time-efficient. Therefore, exploration into the correlation between speech spectrum and various personality traits will become increasingly meaningful for the assessment of employees and election candidates and the computer simulation of speech. Our results demonstrate that personality attributes may be effectively communicated through human speech, yet further research is needed to determine the direction of this effect and the extent to which others' perceptions and subsequent expectations influence the potential emergence of various personality characteristics.
